# Research on the Thermal Decomposition Reaction Kinetics and Mechanism of Pyridinol-Blocked Isophorone Diisocyanate

**DOI:** 10.3390/ma9020110

**Published:** 2016-02-11

**Authors:** Sen Guo, Jingwei He, Weixun Luo, Fang Liu

**Affiliations:** School of Materials Science and Engineering, South China University of Technology, Guangzhou 510641, China; guos0395@126.com (S.G.); msjwhe@scut.edu.cn (J.H.); 263438220@qq.com (W.L.)

**Keywords:** blocked isocyanate, deblocking temperature, thermal decomposition, reaction kinetics, mechanism function, polyurethane

## Abstract

A series of pyridinol-blocked isophorone isocyanates, based on pyridinol including 2-hydroxypyridine, 3-hydroxypyridine, and 4-hydroxypyridine, was synthesized and characterized by ^1^H-NMR, ^13^C-NMR, and FTIR spectra. The deblocking temperature of blocked isocyanates was established by thermo-gravimetric analysis (TGA), differential scanning calorimetry (DSC), and the CO_2_ evaluation method. The deblocking studies revealed that the deblocking temperature was increased with pyridinol nucleophilicity in this order: 3-hydroxypyridine > 4-hydroxypyridine > 2-hydroxypyridine. The thermal decomposition reaction of 4-hydroxypyridine blocked isophorone diisocyanate was studied by thermo-gravimetric analysis. The Friedman–Reich–Levi (FRL) equation, Flynn–Wall–Ozawa (FWO) equation, and Crane equation were utilized to analyze the thermal decomposition reaction kinetics. The activation energy calculated by FRL method and FWO method was 134.6 kJ·mol^−1^ and 126.2 kJ·mol^−1^, respectively. The most probable mechanism function calculated by the FWO method was the Jander equation. The reaction order was not an integer because of the complicated reactions of isocyanate.

## 1. Introduction

Polyurethane is one of the most widely used engineering materials, which can be efficiently tailored as fibers, elastomers, foams, adhesives, and coatings for designed purposes by chemistry and processing [1]. The isocyanate, as a core material, has been widely studied theoretically and practically. The high reactivity and toxicity of isocyanate do not allow for long storage times and use in one-pack systems [2,3]. The blocked isocyanate is an effective solution to solve these flaws [4].

**Scheme 1:**
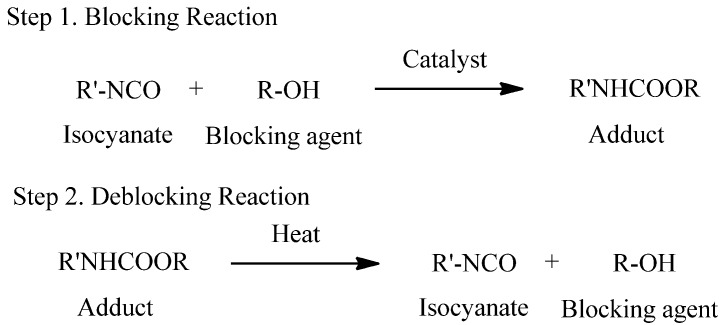
Overall reaction of blocked isocyanates.

A blocked isocyanate is a compound containing relatively weak bond formed by the reaction between an isocyanate and a compound containing an active hydrogen. These adducts are relatively inert at room temperature, but they can regenerate free isocyanates at the deblocking temperature, which can rapidly react with adducts containing the active hydrogen to form more thermally stable bonds [5,6]. The blocking and deblocking reactions are shown in Scheme 1. Blocked isocyanates are preferred for various technical and economic reasons. They have several superiority, such as significant reduction of water sensitivity, elimination of free isocyanate toxicity, and possibility for one-pack and water-borne systems [7].

The research of blocked isocyanate is mostly concentrated on blocking agents, deblocking temperature, and synthesis of blocked isocyanates. The deblocking temperature is an important factor which depends on the structure of isocyanates and blocking agents, the quantity of deblocking catalysts, and the deblocking reaction solvent. Pyridinol was chosen as a blocking agent in this paper because of better hydrophilicity and lower deblocking temperature compared to ethanol and phenol [8].

Meanwhile, there are also a few numbers of articles about the kinetics and mechanisms of blocking-deblocking reaction by using chemical titration [9], infrared spectrum [10], and NMR spectroscopy [11,12]. Two different mechanisms named as “elimination-addition” and “addition-elimination” have been proposed to explain the reaction between blocked isocyanates and nucleophilic adducts. According to the first mechanism, the blocked isocyanate decomposes to produce the free isocyanate, which then reacts with the nucleophilic adducts. According to the second reaction mechanism, the nucleophilic adducts react directly with the blocked isocyanate to form a tetrahedral intermediate. Then, the original blocking agent is eliminated. However, the procedure of the reaction has not been comprehensively studied and the two proposed mechanisms are just applicable in some specific conditions. The thermal decomposition reaction kinetics are rarely reported because of the complexity [13].

In this article we synthesized pyridinol-blocked isophorone diisocyanate and investigated the thermal decomposition reaction kinetics by thermo-gravimetric analysis (TGA) based on the Friedman–Reich–Levi (FRL) equation, the Flynn–Wall–Ozawa (FWO) equation, and the Crane equation. These results may provide some valuable information in theoretical research for the application of blocked isocyanates. 

## 2. Results and Discussion

### 2.1. Synthesis and Characterization of the Blocked Isocyanates

**Figure 1:**
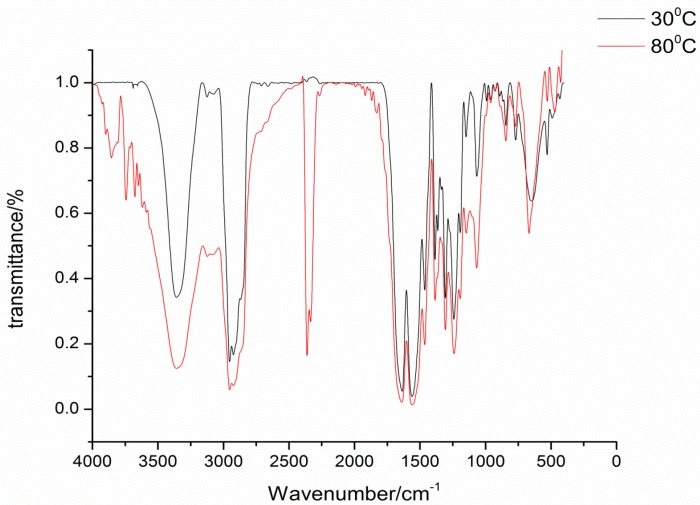
FTIR spectra of blocked isocyanates at different temperatures.

The isophorone diisocyanate was blocked with 2-hydroxypyridine, 3-hydroxypyridine, and 4-hydroxypyridine. The blocking reactions were monitored by FTIR spectroscopy, and the reactions were stopped until the NCO absorption peak at 2270 cm^−1^ completely disappeared. FTIR spectra are successfully used to characterize the blocked diisocyanate. FTIR spectra of the three kinds of synthesized blocked isocyanates are almost the same and show no absorption at the 2270 cm^−1^ range, which indicates that the NCO group of the original isocyanate are completely blocked by pyridinol. In FTIR spectra of 4-hydroxypyridine-IPDI adduct, as an example in Figure 1, the stretching vibration of the C=O group combined with N-H in the urethane absorbs strongly at 1240 cm^−1^. The characteristic absorption frequencies for the N-H stretching at 3346 cm^−1^, C=O stretching at 1693 cm^−1^, and urethane carbamate vibrations at 1562 cm^−1^ indicate that the blocked isocyanate has been synthesized as designed [14]. In Figure 1, it can be easily seen that at 80 °C the NCO group has been successfully regenerated with the absorption at 2270 cm^−1^ range, which proves that the synthesized blocked isocyanates can occur in the deblocking reaction.

Similar to FTIR spectra, ^1^H-NMR and ^13^C-NMR spectra of the synthesized blocked isocyanates are also almost identical. In the ^1^H-NMR spectrum of 4-hydroxypyridine-IPDI adduct in Figure S7, for example, the multiple peaks at 7.1 and 8.3 ppm are due to protons of aromatic rings, and the singlet at 6.7 and 7.6 ppm is due to the proton of the N-H group [15]. The ^13^C-NMR spectrum in Figure S8 has a urethane carbonyl carbon at 156.7 and 159.2 ppm. 

All the characteristic spectra confirm the formation of 4-hydroxypyridine-IPDI adduct. The other two blocked diisocyanates can be characterized by the same methods. The related spectra are shown in Supplementary as Figures S1, S2, S3, S4, S5, S6 and S9, respectively.

### 2.2. Deblocking Temperature 

**Figure 2:**
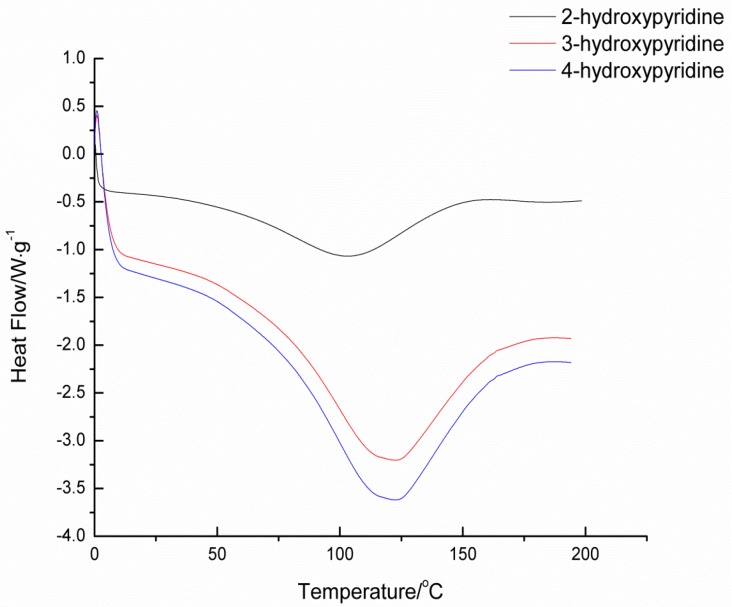
DSC thermograms of blocked isocyanates.

**Figure 3:**
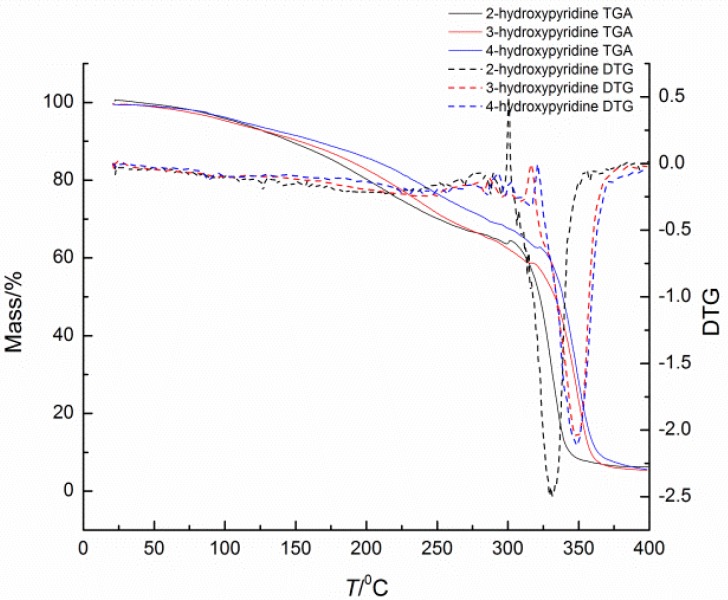
TGA-DTG thermograms of blocked isocyanates.

Deblocking temperature, as an important factor of blocked isocyanates, has been widely studied via many methods. It should be remembered that all the reported deblocking temperatures depend highly on the test methods, heating rates, and many other variables. Thus, the comparisons of deblocking temperatures must be done under the same method and specific condition with extreme care. In this study, the deblocking temperatures of blocked isocyanates were determined by TGA, DSC, and CO_2_ evaluation methods. The deblocking temperatures are listed in Table 1, and the TGA and DSC thermograms of blocked diisocyanates are given in Figure 2 and Figure 3, respectively.

In blocked isocyanates, the urethane bond formed between the isocyanate and the blocking agent is thermally unstable. Therefore there should be an endothermic transition in the DSC curve and weight loss due to the volatilized of the blocking agent in TGA curve. For the same isocyanate structure, the nucleophilicity of the blocking agent is the primary factor of the thermal stability of this weak bond. In this study, the three kinds of blocking agents are just simple pyridinol without substituents and there are no solvents and catalysts during the heating. Thus, the nucleophilicity is mainly affected by the density of electron cloud of pyridine. The deblocking temperature should increase in the order: 3-hydroxypyridine﹥4-hydroxypyridine﹥2-hydroxypyridine, because the relative density of electron cloud increase in that order. The results showed in Table 1 can basically fit the order. 

**Table 1:** Deblocking temperatures of blocked isocyanates.

Blocking Agent	Deblocking Temperature/°C		
	TGA	DSC	CO_2_
2-hydroxypyridine	73	69	76
3-hydroxypyridine	76	72	78
4-hydroxypyridine	71	71	77

### 2.3. Thermal Decomposition Kinetics and Mechanism Functions

**Figure 4:**
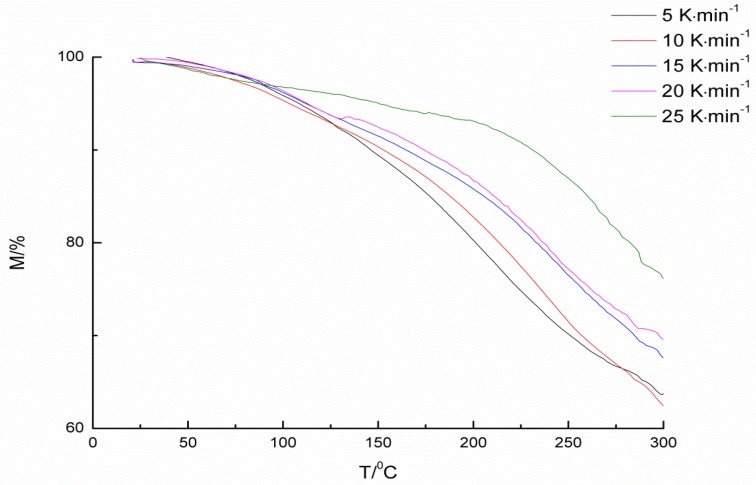
TGA curves of the blocked isocyanate at different heating rates.

Compared with 2-hydroxypyridine and 3-hydroxypyridine, 4-hydroxypyridine has better regularity to make it easier to synthesize multi-substituted pyridinol. Additionally, the deblocking temperatures of three blocked isocyanantes are not much different. 4-hydroxypyridine-IPDI was chosen for further kinetics study. To calculate the kinetics and thermodynamic parameters of the deblocking reaction, non-isothermal experiments were carried out by TGA at different heating rates of 5, 10, 15, 20, and 25 K·min^−1^. The TGA curves are presented in Figure 4.

The basis kinetics data from TGA curves are listed in Table 2. As shown in Figure 4, due to the release of the blocking agent, the extent of thermal decomposition reaction can be tracked by weight loss, which means that the initial temperature could be seen as a deblocking temperature. From the results of Table 2, it can be seen that, as the heating rate rises, the deblocking temperature goes up.

**Table 2:** Basis kinetic data of the blocked isocyanate.

*β*^a^/K·min^−1^	*T*_i_^b^/°C	*T*_p_^c^/°C	Max mass loss/%
5	78	139.7	36.2
10	79	142.2	37.5
15	81	145.5	32.5
20	83	152.4	30.5
25	85	167.6	24.8

Notes: ^a^ *β* is the heating rate; ^b^ *T*_i_ is the initial temperature; ^c^ *T*_p_ is the peak temperature.

Based on the TGA data, Friedman-Reich-Levi (FRL) equation, Flynn–Wall–Ozawa (FWO) equation, and Crane methods are used to calculate the thermal decomposition reaction kinetic of 4-hydroxypyridine blocked isophorone diisocyanate. The details are listed below.

#### 2.3.1. Calculation of Activation Energy (E)

Friedman-Reich-Levi (FRL) equation [16,17] and Flynn–Wall–Ozawa (FWO) [18,19] equation are shown as below:

(1) $$\ln(\frac{\beta d\alpha }{dT})=ln\left[Af(\alpha )\right]-\frac{E}{RT}$$

(2) $$\mathrm{lg}G(\alpha )=\mathrm{lg}\left(\frac{AE}{R}\right)-2.315-\frac{0.4567E}{RT}-\mathrm{lg}\beta$$

where *f(α)* is the differential mechanism function; *G(α)* is the integral mechanism function; *α* is the conversion degree; *T* is the absolute temperature; *A* is the pre-exponential factor; *R* is the gas constant; *E* is the apparent activation energy; and *β* is the heating rate.

**Figure 5:**
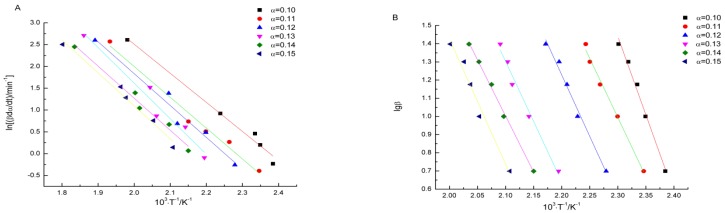
Fitting curves based on FRL (**A**) and FWO (**B**) methods.

By substituting the values of *α*, *β*, and *T* in Table 3 in to the FRL equation, values of the linear correlation coefficient *r*, the slope *b*, and the intercept *a* at different conversion degrees are obtained by the linear least squares method with ln(*βdα*/*dT*) *versus* l*/T*. The activation energy *E* can be calculated from the value of the slope. Meanwhile, in order to assess the value of *E*, the FWO equation is used according to the linear least squares method with lg*β* *versus* l*/T*. All of fitting curves are presented in Figure 5, and calculated results are listed in Table 4.

**Table 3:** Temperatures at the same degree of conversion at different heating rates.

α	T/K				
	β = 5 K·min^−1^	β = 10 K·min^−1^	β = 15 K·min^−1^	β = 20 K·min^−1^	β = 25 K·min^−1^
0.10	419.4	425.7	428.3	431.3	434.6
0.11	426.2	434.9	440.8	444.4	445.9
0.12	438.8	448.7	452.6	455.5	460.5
0.13	455.7	466.9	473.5	475.3	478.3
0.14	465.1	476.9	481.9	487.2	491.4
0.15	474.5	487.0	490.8	493.5	499.6

**Table 4:** Activation energy based on FRL and FWO methods.

α	FRL equation		FWO equation	
	E/kJ·mol^−1^	r	E/kJ·mol^−1^	r
0.10	122.0	0.9832	147.1	0.9949
0.11	129.4	0.9821	118.6	0.9969
0.12	133.8	0.9837	123.2	0.9937
0.13	149.4	0.9809	120.5	0.9817
0.14	134.7	0.9909	122.4	0.9888
0.15	138.2	0.9808	125.5	0.9888

Based on the FRL method, the average of activation energy is 134.6 kJ·mol^−1^ with a relative standard deviation of 6%. The average of activation energy calculated by FWO method is 126.2 kJ·mol^−1^ with a relative standard deviation of 8%. The activation energy values calculated by these two methods are close to each other, and they both have little variation with the changes of the conversion degree. All of the linear correlation coefficients *r* approach 1, which means the fitting curves were dependable [20].

#### 2.3.2. Determination of F(α) and G(α)

By substituting the values of conversion degrees at the same temperature on several TGA curves, the different mechanism functions *G(α)*, and various heating rates in Equation (2), values of the linear correlation coefficient *r*, the slope *b*, and the intercept *a* at different temperatures can be obtained by the linear least squares method with lg*G*(*α*) *versus* lg*β*. If the linear correlation coefficient *r* is the best and the slope *b* approaches −1, the relevant function is the probable mechanism function of a solid-phase reaction [21]. Since there are more than 30 conversion functions [22] to be calculated, only a part of the results is shown in Table 5 as an example.

**Table 5:** Part of the results from the linear least squares method at different kinetic mechanisms of thermal decomposition.

T/K	Function	*b*	*r*
	Valensi	−0.6721	0.9231
403.2	Jander	−0.9879	0.9941
	Mampel Power	−0.7258	0.9152
	Valensi	−0.4315	0.8956
423.2	Jander	−0.9731	0.9965
	Mampel Power	−0.6532	0.9972
	Valensi	−0.7635	0.9466
443.2	Jander	−1.0245	0.9895
	Mampel Power	−2.0615	0.9358
	Valensi	−0.8527	0.9911
463.2	Jander	−0.9851	0.9953
	Mampel Power	−1.4627	0.9512
	Valensi	−0.7855	0.9263
483.2	Jander	−0.9758	0.9910
	Mampel Power	−1.1284	0.9599

It can be easily seen from Table 4 that only the linear correlation coefficient *r* of the Jander equation is the best and the slope *b* approaches −1 at five different temperatures. Therefore it can be summarized that the most probable mechanism function is $G(\alpha )={\left[1-{\left(1+\alpha \right)}^{1/3}\right]}^{1/2}$ and $f(\alpha )=6{\left(1-\alpha \right)}^{2/3}{\left[1-{\left(1+\alpha \right)}^{1/3}\right]}^{1/2}$.

#### 2.3.3. Calculation of the Reaction Order (n)

The Crane equation is shown as follows [23]:

(3) $$\frac{d\ln\beta }{d\left(1/T_{p}\right)}=-\frac{E}{nR}-2T_{p}$$

where *n* is the reaction order.

When $E/nR\gg 2T_{p}$ $E/nR\gg 2T_{p}$, Equation (3) can be simplified as follows:

(4) $$\frac{d\ln\beta }{d\left(1/T_{p}\right)}=-\frac{E}{nR}$$

By substituting the values of temperatures at the same degree of conversion, heating rates in Equation (4), values for the linear correlation coefficient *r*, the slope *b*, and the intercept *a* at different conversion degrees can be obtained by the linear least squares method with ln*β versus* l/*T*. From the value of the slope and activation energy, the reaction order can be calculated [24]. The reaction order *n* can be calculated from the value of the slope with the average activation energy *E* calculated by FRL and FWO method. The results are listed in Table 6.

**Table 6:** Reaction order based on the Crane equation.

α	n	r
0.10	1.25	0.9974
0.11	1.23	0.9889
0.12	1.28	0.9934
0.13	1.24	0.9888
0.14	1.27	0.9961
0.15	1.25	0.9983

From Table 6, it can be seen that all the reaction orders *n* are not integers because of the complicated reaction of isocyanates [25]. Even heating a blocked isocyanate without nucleophile and catalyst, as the simplest case, is accompanied with reversible reactions and possible side reactions of the highly active isocyanate. At high temperatures, there are dimerization or trimerization reactions of isocyanate, especially for aromatic isocyanate, and reactions between the regenerated isocyanate and original blocked isocyanate to form an allophanate or biuret [26]. The blocked isocyanate can also thermally decompose through different mechanisms at different temperatures [27]. In some cases, the deblocking reaction may be catalyzed by blocked isocyanate itself or the regenerated blocking agent.

However, the FRL and FWO methods ignore the complicated thermal decomposition reaction and focus on the energy change during the thermal decomposition reaction, which makes the related kinetic data and probable mechanism function believable [22].

## 3. Experimental Section

### 3.1. Materials

2-hydroxypyridine, 3-hydroxypyridine, 4-hydroxypyridine, isophorone diisocyanate, and dibutyltin dilaurate were purchased from J and K Scientific Ltd. (Shanghai, China) and purified before used. Methylbenzene and petroleum ether (boiling point between 60 and 80 °C) were obtained by Guangzhou Reagent Co. (Guangzhou, China), and distilled under vacuum before use.

### 3.2. Synthesis of Pyridinol Blocked Isophorone Diisocyanate

In a typical synthesis, 30 ml of 2-hydroxypyridine methylbenzene solution (2.0 M) and dibutyltin dilaurate (DBTDL, 0.5% by weight of isocyanate) were added into a dry three-necked flask equipped with a condenser, a dropping funnel, and a nitrogen gas inlet. Then 50 mL of isophorone diisocyanate methylbenzene solution (1.0 M) was added in the dropping funnel and dropped into one neck of the adding petroleum ether into the reaction mixture and dried in vacuum at 40 °C with a yield of 65%.

### 3.3. Characterization of Blocked Isocyanates

^1^H-NMR and ^13^C-NMR spectrum of the product was measured by an Avance III HD 400MHz Instrument (Bruker Co., Karlsruhe, Germany) with deuterated chloroform (DMSO-d6) as solvent and tetramethylsilane (TMS) as an internal reference [29]. FTIR spectrum for the products was recorded by the potassium bromide pellet method at room temperature in a Vector33 Model Fourier Transform Infrared Instrument (Bruker Co., Germany). [29] The sample was scanned 32 times between 400 and 4000 cm^−1^ with circulation of 4 cm^−1^.

2-hydroxypyridine-IPDI adduct: ^1^H NMR (400 MHz, DMSO-d6, δ): 7.6, 7.2, 6.5, 6.4 (8H, Ar H), 7.6, 6.7(2H, NH), 4.0 (1H, C-H), 2.7, 3.1 (2H, CH2NH), 1.2-1.5 (6H, CH2), 0.7-1.0 (9H, CH3). ^13^C-NMR (400 MHz, DMSO-d6, δ): 160.3, 147.2, 142.4, 125.1, 114.3 (Ar C), 157.2, 155.1 (C=O), 50.7 (CH), 49.1 (CH2NH), 48.8, 46.6, 44.6 (CH2), 31.4, 26.4 (CH3). IR (KBr): ν = 3341 (w, N-H), 2959 (w, C-H), 1691 (s, C=O), 1567 (s, urethane).

3-hydroxypyridine-IPDI adduct: ^1^H NMR (400 MHz, DMSO-d6, δ): 8.2, 8.1, 7.3 (m, 8H, Ar H), 7.6, 6.7(s, 2H, NH), 4.0 (m, 1H, C-H), 2.7, 3.1 (m, 2H, CH2NH), 1.2-1.5 (m, 6H, CH2), 0.7-1.0 (m, 9H, CH3). ^13^C-NMR (400 MHz, DMSO-d6, δ): 158.1, 156.1 (C=O), 145.8, 144.3, 143.2, 130.4, 125.1 (Ar C), 50.7 (CH), 49.1 (CH2NH), 48.8, 46.6, 44.6 (CH2), 31.4, 26.4 (CH3). IR (KBr): ν = 3351 (w, N-H), 2952 (w, C-H), 1693 (s, C=O), 1567 (s, urethane).

4-hydroxypyridine-IPDI adduct: ^1^H NMR (400 MHz, DMSO-d6, δ): 8.3, 7.1 (m, 8H, Ar H), 7.6, 6.7(s, 2H, NH), 4.0 (m, 1H, C-H), 2.7, 3.1 (m, 2H, CH2NH), 1.2-1.5 (m, 6H, CH2), 0.7-1.0 (m, 9H, CH3). ^13^C-NMR (400 MHz, DMSO-d6, δ): 161.3, 149.2, 118.6 (Ar C), 159.2, 156.7 (C=O), 50.7 (CH), 49.1 (CH2NH), 48.8, 46.6, 44.6 (CH2), 31.4, 26.4 (CH3). IR (KBr): ν = 3346 (w, N-H), 2950 (w, C-H), 1693 (s, C=O), 1562 (s, urethane).

### 3.4 Assessment of deblocking temperature

#### 3.4.1. TGA Testing

Thermo-gravimetric analysis (TGA) has been used to determine kinetic parameters for deblocking reactions. The extent of reaction is followed by tracking weight loss due to the release of the blocking agent. The TGA curves were obtained with TGA7 thermo-gravimetric analyzer (Perkin Elmer Co., Phoenix, Arizona, USA) under a nitrogen atmosphere. The temperature was increased from room temperature to 250 °C with heating rates of 5, 10, 15, 20, and 25 K·min^−1^. The weight of the sample was approximately 6.0 to 7.0 mg.

#### 3.4.2. DSC Testing

The changes in heat flow associated with the deblocking reaction, as measured by differential scanning calorimetry (DSC), have been used to determine the deblocking temperature. The DSC curves were obtained with a Q20 differential scanning calorimeter (TA Co., New Castle, Pennsylvania, USA) under a nitrogen atmosphere. The temperature was increased from room temperature to 250 °C with a heating rate of 15 K·min^−1^. The weight of sample was approximately 6.0 to 7.0 mg.

#### 3.4.3. CO_2_ Evaluation Method

The CO_2_ evaluation method was used to measure the deblocking temperatures of blocked isocyanate. In a typical experiment, 0.5–0.6 g of blocked isocyanates was dissolved in 20 mL of DMF together with 5 g water at 30 °C in a three-neck flask equipped with a dry, carbon dioxide-free nitrogen inlet and another neck was connected to a saturated solution of barium hydroxide. The flask was heated in a silicone oil bath at a rate of 3 °C·min^−1^. As deblocking occurs, regenerated NCO reacts with the water, liberating CO_2_. Then the reaction between CO_2_ and barium hydroxide causes turbidity in the saturated solution of barium hydroxide. The deblocking temperature was taken as the minimum temperature when the turbidity appeared.

## 4. Conclusions

A series of pyridinol-blocked isophorone isocyanates is synthesized and characterized. The deblocking temperature is increased with pyridinol nucleophilicity based on DSC, TGA, and CO_2_ evolution method. The thermo-gravimetric analysis is used to study the deblocking temperature of the synthesized blocked diisocyanate. The deblocking temperature has a shift to higher temperature with an increase in heating rate. The Friedman–Reich–Levi (FRL) equation and Flynn–Wall–Ozawa (FWO) equation can be applied to analyze the thermal decomposition reaction of blocked isocyanates, and both the linear correlation coefficient are good enough to use as described. The calculated activation energy is 134.6 kJ·mol^−1^ and 126.2 kJ·mol^−1^ according to FRL method and FWO method, respectively. The most probable mechanism function calculated by FWO method is Jander equation. The function is $G(\alpha )={\left[1-{\left(1-\alpha \right)}^{1/3}\right]}^{1/2}$ and $f(\alpha )=6{\left(1-\alpha \right)}^{2/3}{\left[1-{\left(1-\alpha \right)}^{1/3}\right]}^{1/2}$. The reaction order is not an integer because of the complicated thermal decomposition reaction.

## Supplementary Files

Supplementary File 1
