# Supplementary material for: Research on the Thermal Decomposition Reaction Kinetics and Mechanism of Pyridinol-Blocked Isophorone Diisocyanate
Source: Materials (Basel). 2016 Feb 11;9(2):110. doi: 10.3390/ma9020110 (PMC5456470; doi:10.3390/ma9020110)
Supplement: Supplementary File 1 [file materials-09-00110-s001.docx]

**Supplementary Materials: Research on the Thermal Decomposition Reaction Kinetics and Mechanism of Pyridinol-Blocksed Isophorone Diisocyanate**

Sen Guo, Jingwei He, Weixun Luo and Fang Liu


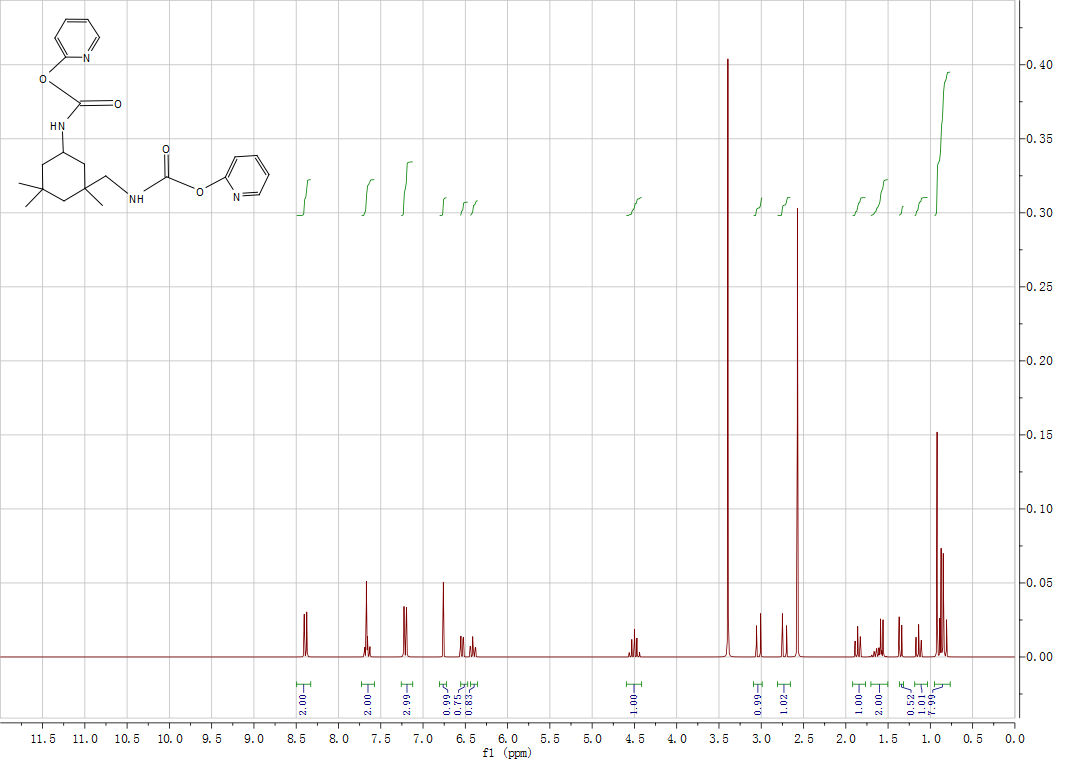


**Figure S1.** ^1^H NMR spectrum of 2-hydroxypyridine-IPDI adduct.


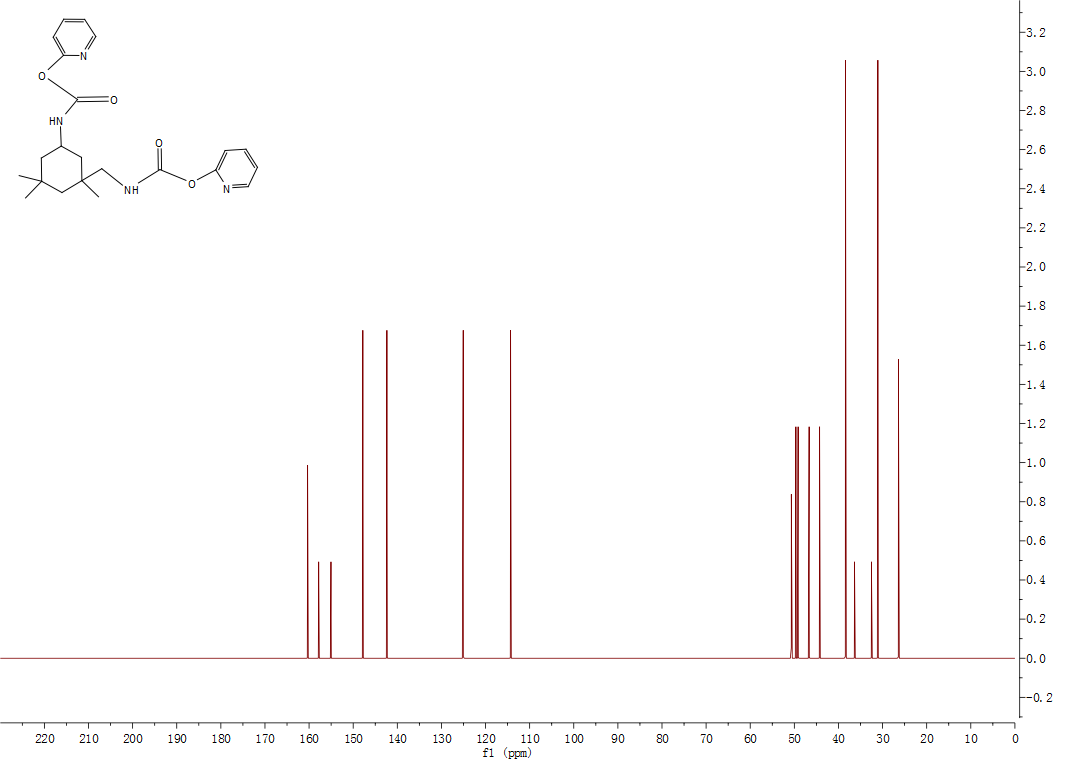


**Figure S2.** ^13^C-NMR spectrum of 2-hydroxypyridine-IPDI adduct.





**Figure S3.** FTIR spectrum of 2-hydroxypyridine-IPDI adduct


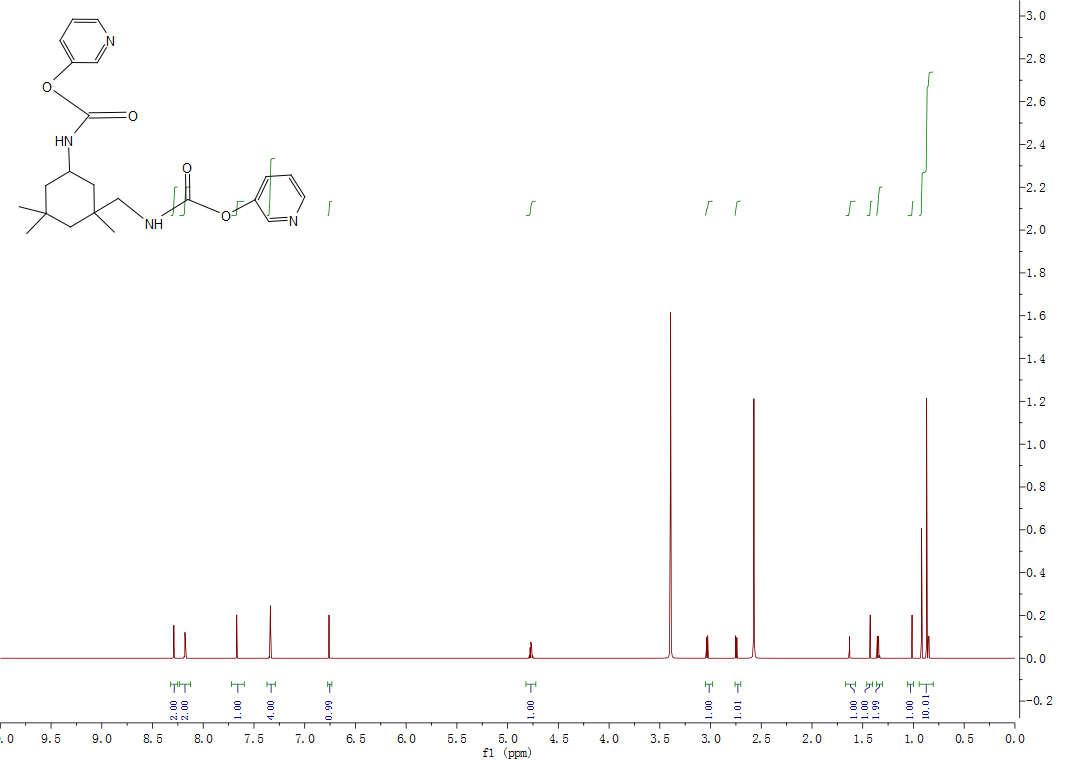


**Figure S4.** 1H NMR spectrum of 3-hydroxypyridine-IPDI adduct.


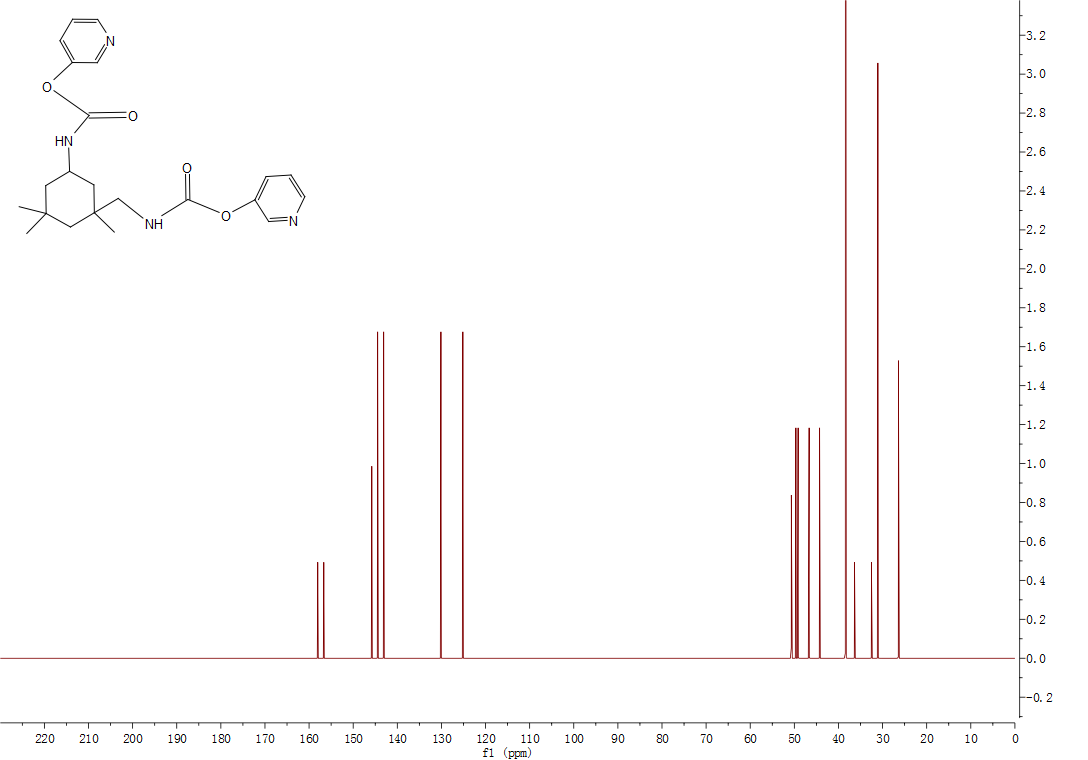


**Figure S5**. 13C-NMR spectrum of 3-hydroxypyridine-IPDI adduct.





**Figure S6**. FTIR spectrum of 3-hydroxypyridine-IPDI adduct.


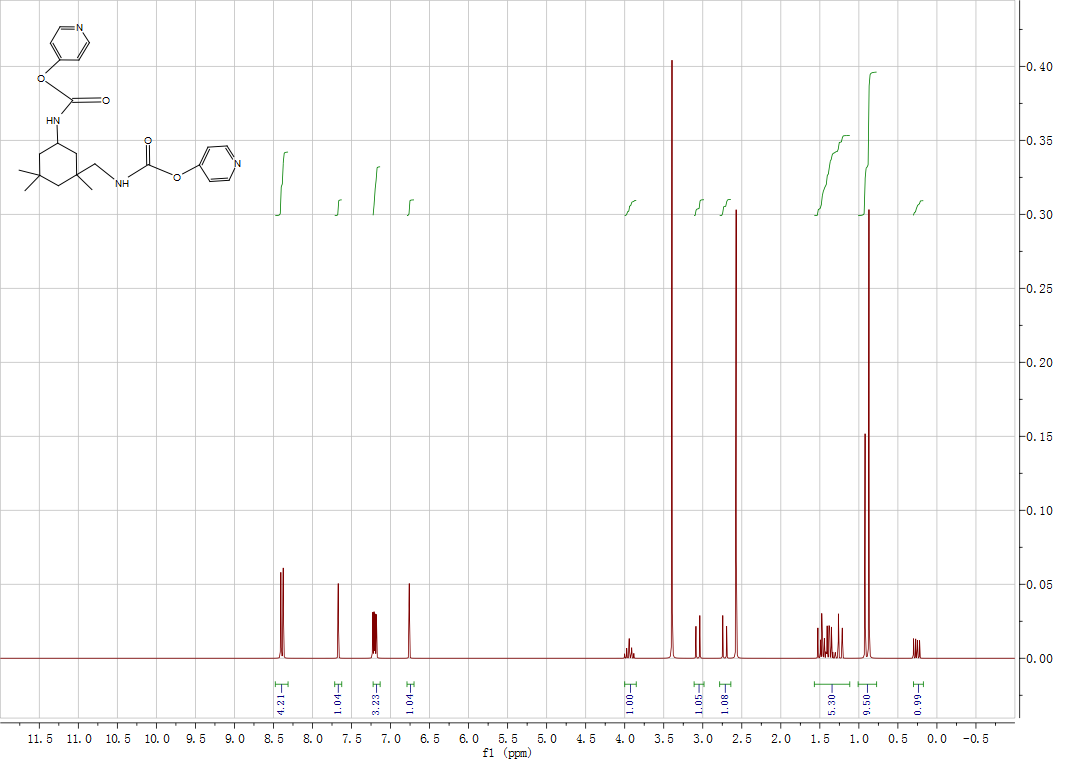


**Figure S7**. 1H NMR spectrum of 4-hydroxypyridine-IPDI adduct.


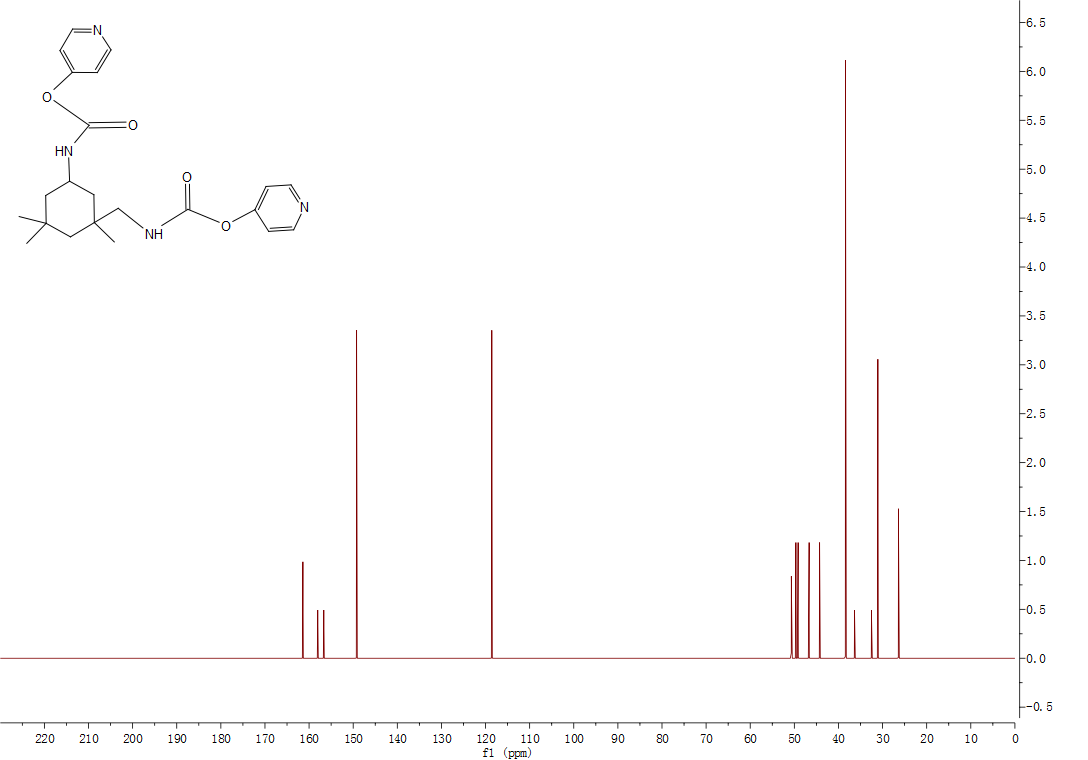


**Figure S8**. 13C-NMR spectrum of 4-hydroxypyridine-IPDI adduct.





**Figure S9**. FTIR spectrum of 4-hydroxypyridine-IPDI adduct.

© 2016 by the authors; licensee MDPI, Basel, Switzerland. This article is an open access article distributed under the terms and conditions of the Creative Commons by Attribution (CC-BY) license (http://creativecommons.org/licenses/by/4.0/).
